# Prospective study on smoking cessation duration and wound healing outcomes in reconstructive surgery

**DOI:** 10.6026/973206300213227

**Published:** 2025-09-30

**Authors:** Brinda Ganesh Kolar, Yeruva Bheemeswara Reddy, Dhyaneshwar Ra, Vishal Mruthyunjaya Kalmani, Poornima Venkatraman

**Affiliations:** 1 Department of Cardiology, University Hospitals Bristol and Weston, South West England, United Kingdom; 2 Department of Surgery, Santhiram Medical College andhra Pradesh, India; 3 Department of General Surgery, Government Stanley Medical College Chennai, Tamil Nadu, India; 4 Department of Surgery, Barking Havering and Redbridge University Hospitals NHS Trust, Essex, United Kingdom; 5 Department of Surgery, Davao Medical School Foundation, Philippines

**Keywords:** Smoking cessation, wound healing, reconstructive surgery, wound complications, preoperative care

## Abstract

The relationship between the duration of smoking cessation before reconstructive surgery and subsequent wound healing outcomes is of interest. Hence, a total of 128 adult patients undergoing elective reconstructive procedures were followed for 30 days postoperatively. Wound complications-including infection, dehiscence and delayed healing-were assessed in relation to smoking abstinence duration. Patients who ceased smoking for ≥4 weeks showed significantly better healing outcomes than those who quit for <2 weeks. Thus, we show the clinical importance of extended preoperative smoking cessation for optimized wound recovery.

## Background:

Smoking is a well-established risk factor for impaired wound healing, particularly in patients undergoing reconstructive surgery where tissue viability and healing integrity are critical [1]. Nicotine and other toxic compounds in tobacco smoke led to vasoconstriction, reduced tissue oxygenation, impaired collagen synthesis and diminished immune response-all of which contribute to increased rates of wound dehiscence, infection and delayed healing [2]. Numerous studies have demonstrated the adverse effects of active smoking on surgical outcomes; however, there remains variability in clinical guidelines regarding the optimal duration of preoperative smoking cessation required to reduce such risks [3]. Despite growing awareness, many patients struggle to quit smoking or underestimate the time needed for physiological recovery following tobacco use [4]. Evidence suggests that even short-term cessation may confer some benefit, but the degree of risk reduction appears to be closely tied to the length of abstinence prior to surgery [5]. Therefore, it is of interest to prospectively evaluate how varying durations of smoking cessation influence postoperative wound healing outcomes in patients undergoing elective reconstructive procedures. By identifying a clear threshold for preoperative abstinence, the findings may contribute to more definitive clinical recommendations and enhanced perioperative counselling strategies.

## Methods and Methods:

This prospective observational study was conducted at a tertiary care centre between January 2024 and December 2024. A total of 128 adult patients scheduled for elective reconstructive surgery were enrolled after providing informed consent. Inclusion criteria comprised patients aged 18-65 years with a history of active smoking (minimum 5 cigarettes/day for at least 1 year) who had voluntarily ceased smoking prior to surgery. Patients were stratified into three groups based on self-reported duration of preoperative smoking cessation: <2 weeks, 2-4 weeks and >4 weeks. All surgeries were performed by the same surgical team to minimize procedural variability. Standardized postoperative wound care protocols were followed and patients were monitored for 30 days postoperatively for signs of wound complications, including surgical site infection, dehiscence, necrosis, or delayed healing. Wound assessment was conducted by blinded evaluators using objective clinical criteria. Data on demographic variables, comorbidities, type and duration of surgery and compliance with smoking cessation were also collected. Statistical analysis included chi-square tests and logistic regression to determine the association between smoking cessation duration and wound outcomes, with a significance level set at p < 0.05.

## Results:

A total of 128 patients were analysed, stratified into three smoking cessation duration groups: <2 weeks (n=43), 2-4 weeks (n=41) and >4 weeks (n=44). Patients with longer durations of smoking cessation experienced significantly fewer wound complications, shorter healing times and better postoperative outcomes compared to those with minimal cessation periods. Table 1 (see PDF) shows patients who ceased smoking for longer durations were generally older and had higher BMI compared to short-term quitters, although differences were not statistically significant. Table 2 (see PDF) shows a significantly higher proportion of patients in the <2 weeks cessation group developed wound complications compared to those in the >4 weeks group. Table 3 (see PDF) shows patients who quit smoking for >4 weeks had a significantly faster mean wound healing time compared to the other groups. Table 4 (see PDF) shows longer cessation was associated with fewer overall postoperative complications. Table 5 (see PDF) shows smoking cessation duration was an independent predictor of wound complications even after adjusting for confounding variables. Table 6 (see PDF) shows wound infection was the most common complication in those with <2 weeks of smoking cessation. Table 7 (see PDF) shows surgical site location did not significantly affect the rate of complications. Table 8 (see PDF) shows use of perioperative antibiotics was consistent across groups and did not confound wound healing results. Table 9 (see PDF) shows patient-reported wound satisfaction scores were significantly higher in the >4 weeks cessation group. Table 10 (see PDF) shows longer cessation was associated with shorter hospital stays post-surgery.

## Discussion:

The findings of this prospective study clearly demonstrate that longer durations of preoperative smoking cessation are associated with significantly improved wound healing outcomes following reconstructive surgery [6]. Patients who ceased smoking for more than 4 weeks experienced notably lower rates of wound infections, dehiscence, delayed healing and other postoperative complications compared to those who quit for less than 2 weeks [7]. These results align with existing literature suggesting that physiological recovery from the effects of smoking-such as improved tissue oxygenation, collagen synthesis and immune function-requires several weeks of abstinence to become clinically significant [8]. Moreover, logistic regression confirmed smoking cessation duration as an independent predictor of wound complications, even after adjusting for age, BMI, diabetes and other factors [9]. While baseline characteristics such as age and comorbidities were relatively balanced across groups, the consistent trends in healing time, satisfaction scores and hospital stay duration further support the protective role of extended smoking cessation [10]. Interestingly, the total years of smoking history did not significantly impact complication rates within cessation groups, suggesting that recent behavioural change may outweigh long-term smoking exposure in short-term surgical outcomes [11]. These findings underscore the necessity of structured preoperative counselling and smoking cessation programs, emphasizing a minimum 4-week abs tine nice window wherever feasible. Such efforts could substantially reduce postoperative morbidity, healthcare costs and readmissions [12]. Limitations include the reliance on self-reported smoking cessation and lack of biochemical verification, which may introduce bias. Nevertheless, the study reinforces the importance of proactive perioperative tobacco control as a modifiable risk factor in surgical wound management.

## Conclusion:

Preoperative smoking cessation for more than 4 weeks significantly improves wound healing outcomes in reconstructive surgery. Patients with shorter cessation durations face higher complication rates and prolonged recovery. Structured cessation protocols should be prioritized in surgical planning to enhance postoperative outcomes.
